# Role of oxidative stress on diesel-enhanced influenza infection in mice

**DOI:** 10.1186/1743-8977-7-34

**Published:** 2010-11-22

**Authors:** Kymberly M Gowdy, Quentin T Krantz, Charly King, Elizabeth Boykin, Ilona Jaspers, William P Linak, M Ian Gilmour

**Affiliations:** 1 Division of Pulmonary, Allergy, and Critical Care, Duke University Medical Center, 106 Research Dr., 2100B MSRB2, Durham, NC 27710, USA; 2 Environmental Public Health Division, National Health and Environmental Effects Research Laboratory, United States Environmental Protection Agency, 109 T.W. Alexander Dr., RTP, NC, 27711, USA; 3 Air Pollution Prevention and Control Division, National Risk Management Research Laboratory, United States Environmental Protection Agency, 109 T.W. Alexander Dr., RTP, NC 27711, USA; 4 Center for Environmental Medicine, Asthma, and Lung Biology, University of North Carolina, 104 Mason Farm Rd, CB 7310, Chapel Hill, NC 27599, USA; 5 Department of Pediatrics, University of North Carolina, 104 Mason Farm Rd, CB 7310, Chapel Hill, NC 27599, USA

## Abstract

Numerous studies have shown that air pollutants, including diesel exhaust (DE), reduce host defenses, resulting in decreased resistance to respiratory infections. This study sought to determine if DE exposure could affect the severity of an ongoing influenza infection in mice, and examine if this could be modulated with antioxidants. BALB/c mice were treated by oropharyngeal aspiration with 50 plaque forming units of influenza A/HongKong/8/68 and immediately exposed to air or 0.5 mg/m^3 ^DE (4 hrs/day, 14 days). Mice were necropsied on days 1, 4, 8 and 14 post-infection and lungs were assessed for virus titers, lung inflammation, immune cytokine expression and pulmonary responsiveness (PR) to inhaled methacholine. Exposure to DE during the course of infection caused an increase in viral titers at days 4 and 8 post-infection, which was associated with increased neutrophils and protein in the BAL, and an early increase in PR. Increased virus load was not caused by decreased interferon levels, since IFN-β levels were enhanced in these mice. Expression and production of IL-4 was significantly increased on day 1 and 4 p.i. while expression of the Th1 cytokines, IFN-γ and IL-12p40 was decreased. Treatment with the antioxidant N-acetylcysteine did not affect diesel-enhanced virus titers but blocked the DE-induced changes in cytokine profiles and lung inflammation. We conclude that exposure to DE during an influenza infection polarizes the local immune responses to an IL-4 dominated profile in association with increased viral disease, and some aspects of this effect can be reversed with antioxidants.

## Introduction

Viral infections are a major cause of pulmonary-related illnesses in children, the elderly, and other susceptible populations such as asthmatics [1-3]. Epidemiological studies have noted an association between air pollution exposure and an increased rate of pulmonary infections [4,5]. Laboratory research has also shown that exposure to airborne particulate matter (PM) increases susceptibility to both bacterial and viral pathogens (reviewed in [6]). Diesel exhaust (DE) is a significant contributor to urban air pollution and has been shown to increase susceptibility to infections [7-13] although the mechanisms that underlie this process are not fully understood. Several laboratories have demonstrated that rodents exposed to high concentrations of re-suspended diesel exhaust particles (DEP) have impaired clearance of gram negative and gram positive bacteria as a result of reduced phagocytosis [14-16]. Exposure to lower concentrations of fresh DE has also been shown to increase susceptibility to respiratory syncytial virus (RSV) and influenza infection [10,11]. These reports only examined how DE altered the pulmonary environment prior to infection however, and did not consider the immunomodulatory effects of DE exposure during viral illness.

Influenza is a respiratory virus that accounts for approximately 36,000 deaths and over 100,000 hospitalizations each year despite large-scale vaccination and antiviral treatment [17,18]. Influenza replicates primarily in the epithelial cells of the respiratory tract, but can also infect macrophages and monocytes. The clearance of influenza relies on the production by multiple cells of anti-viral type I interferons and Th1 cytokines [19], while the Th2 cytokine IL-4, delays the recovery from viral infection [20-22].

Animal and human *in vitro *and *in vivo *studies have shown that exposure to DE increases neutrophil recruitment, nitric oxide production, and pro-inflammatory cytokines [23-29]. DE alone or in the context of antigen exposure also increases expression of the Th2 cytokines IL-4 and IL-13, while decreasing expression of the Th1 cytokine IFN-γ [30-32]. Exposure to DE causes oxidative stress in target cells [28,33] through development of reactive oxygen species (ROS) that induce the transcription of phase II enzymes including heme-oxygenase 1 (HO-1) and catalase [33,34]. ROS interfere with the polarity of the immune response through depletion of glutathione in DCs, which downregulates IL-12 production and increases IL-4, favoring a Th2 phenotype [35].

Since most reports have examined the effect of DE on subsequent immune responses to pathogens or antigens, the present study was designed to address how DE affected development of protective immune responses to an ongoing infection in mice. Because DE is known to promote Th2 cytokine production and, IL-4 is known to delay viral clearance, we hypothesized that the DE exposure would enhance the development of viral disease through IL-4 production and promotion of a Th2 phenotype while causing a concomitant dampening of Th1 protective immunity. In addition it was of interest to determine whether an antioxidant could mitigate this effect thus providing potential strategies for reducing the health impact of air pollution-enhanced respiratory infections.

## Materials and methods

### Animals

Pathogen-free BALB/c female mice, 10-12 wk old, weighing 17-20 g, were purchased from Charles River (Raleigh, NC). Once at the U.S. EPA animal care facilities (accredited by the Association for Assessment and Accreditation of Laboratory Animal Care), animals were housed in groups of five in polycarbonate cages with hardwood chip bedding (Beta Chip, Northeastern Products, Warrensburg, NY), provided a 12-hour light (0600 hours) to dark (1800 hours) cycle, maintained at 22.3 ± 1.1°C and 50 ± 10% humidity, and given access to both food (5P00 Prolab RMH 3000, PMI Nutrition International, Richmond, IN) and water *ad libitum*. Animals were acclimated for at least ten days before the study began. Sentinel animals were housed in the same location and found to be free of common rodent pathogens. The first study was repeated in its entirety and then a third experiment was designed to reproduce noted effects and examine how anti-oxidant treatment affected the outcome. All procedures were approved by the laboratory's Institutional Animal Care and Welfare Committee.

### Influenza Virus

The influenza A/HongKong/8/68 (H3N2 serotype) used in this study was obtained from Dr. Dori Germolec (Laboratory of Respiratory Biology, NIEHS, NIH, RTP, NC 27709). The virus was used to prepare dilutions in sterile saline containing 50 PFUs in 50 μl. Virus titers were determined using influenza infection of Madin-Darby canine kidney cells. Stock virus was aliquoted and stored at -80°C until use.

### Oropharyngeal Aspiration of Virus

Immediately before the first DE inhalation exposure, mice were anesthetized in a small plexiglass box using vaporized isofluorane (Webster Veterinary Supply Inc., Sterling, MA). Anesthetized mice were suspended vertically by their front incisors on a small wire attached to a support. The tongue was extended with forceps and 50 μl of either sterile saline (Hospira Inc., Lake Forest, IL) or 50 plague forming units (PFUs) (10^5.3 ^TCID_50_, LD_50 _is 200 PFUs) of influenza A/Honkong/8/68 (H3N2 serotype) was instilled into the oro-pharynx using a 1 ml syringe fitted with a 24 gauge intragastric feeding needle, with a 1.25 mm-diameter ball tip. The nose of the mouse was then covered, causing the liquid to be aspirated into the lungs.

### Diesel Exhaust Exposure and Monitoring

Diesel exhaust for animal inhalation exposure experiments was generated using a 134 kW (180 hp) 8-cylinder 6.5 liter displacement indirect injection Detroit Diesel engine mounted in a 1994 Chevrolet Cheyenne 2500 pickup truck equipped with a manual transmission and oxidation catalyst. The engine and transmission were connected directly to a Land & Sea (model DYNOmite 300) eddy current dynamometer to provide a load. The equipment was operated in an attempt to simulate steady-state highway operation. The engine and transmission were operated at 2500 rpm in third (1:1 ratio) gear. The dynamometer was operated at 7 amps, providing approximately 100 ft/lbs of torque after a warm-up period. The projected load was equivalent to approximately 25% of the maximum engine load (at 2500 rpm). The truck speedometer (measuring drive shaft rpm) indicated a steady speed of 55 miles/h. Road taxed diesel fuel was purchased from a local (Research Triangle Park, NC) service station and stored in 55 gal drums. Replicate analysis (ultimate, elemental, heating value, and specific gravity) of multiple batches of fuel purchased over time indicated consistent fuel properties and composition (data not shown). Engine lubrication oil (Shell Rotella, 15W-40) was changed before each set of exposure tests.

From the engine exhaust, a small portion of the flow (14 L/min) was educted by an aspirator (3:1 dilution) to a second cone diluter (10:1 dilution), and then through approximately 10 meters of stainless steel tubing (7 cm inside diameter) to a stainless steel Hazelton (model 1000) exposure chamber housed in an isolated animal exposure room. The dilution air used was drawn from the animal exposure room through an activated carbon bed and high efficiency particulate air (HEPA) filter. The target diesel emission particle (DEP) concentration in the chamber (500 μg/m^3^) was continuously monitored using a tapered element oscillating microbalance (TEOM, Rupprecht and Patashnick Co., series 1400, Albany, NY). Dilution air was periodically adjusted to control the DEP concentrations. Control animals were housed in a separate chamber supplied with the same activated carbon/HEPA filtered room air. The two diesel chambers (one containing influenza infected mice and the other sham saline controls) were operated at the same flow rate (280 L/min), resulting in 16 full air exchanges per hour.

Integrated 4 h filter samples (14.1 L/min) were collected daily from each chamber and analyzed gravimetrically to determine particle concentrations. In addition, 20-min quartz filter samples (14.1 L/min) were collected from the DEP exposure chamber each day and analyzed using a thermal/optical carbon analyzer (Sunset Laboratory Inc., model 107, Tigard, OR) to determine organic carbon/elemental carbon (OC/EC) partitioning of the collected DEP. TEOM measurements, continuous emission monitors (CEMs) were used to measure chamber concentrations of oxygen (O_2, _Beckman Corp., model 755, La Habra, CA), carbon monoxide (CO, Thermo Electron Corp, model 48, Franklin, MA), nitrogen oxides (NO_x_, Teledyne Technology Co., model 200A4, San Diego, CA), and sulfur dioxide (SO_2, _Thermo Electron Corp, model 43 c, Franklin, MA). Samples were extracted through fixed stainless steel probes in the exposure chambers. Gas samples were passed through a particulate filter prior to the individual gas analyzers. Particle size distributions were characterized during each exposure using a scanning mobility particle sizer (SMPS, TSI Inc., model 3080/3022a, St. Paul, MN) and an aerodynamic particle sizer (APS, TSI Inc., model 3321, St. Paul, MN). Chamber temperatures, relative humidity, and noise were also monitored, and maintained within acceptable ranges. Mice were exposed to DEP or filtered air for 4 h/day for 13 consecutive days. The study was repeated in full and the experimental data were combined.

### Bronchoalveolar Lavage

On day 1, 4, 8, and 14 post-infection (p.i.) mice from each treatment group were euthanized with sodium pentobarbital and the trachea was exposed, cannulated, and secured with suture thread. The left mainstem bronchus was then isolated, clamped with microhaemostats after the trachea was cannulated. The right lung lobes were lavaged 3 times with a single volume of warmed Hanks balanced salt solution (HBSS) (Invitrogen, Grand Island, NY) at a rate of 35 ml/kg. The resulting lavage was centrifuged (717 × g, 15 min, 4°C) and stored at -80°C for cytokine measurement or at 4°C for protein measurement. The pelleted cells were resuspended in 1 ml of RPMI 1640 (Gibco, Carlsbad, CA) containing 2.5% fetal bovine serum (FBS; Gibco, Carlsbad, CA). Total cell counts in the lavage fluid of the right lobe were obtained with a Coulter Counter (Beckman Dickson, Fullerton, CA). Each sample (200 μl) was centrifuged in duplicate onto slides using a Cytospin (Shandon, Pittsburgh, PA) and subsequently stained with Diff Quik solution (American Scientific, McGraw Park, PA) for differential cell counts, with at least 200 cells counted from each slide. The left lobe was snap frozen in liquid nitrogen and subsequently stored at -80°C for isolation of RNA and protein or homogenized in 250 μl of DMEM containing 1 μg/ml of TPCK treated trypsin and 1% BSA for viral titers.

### BAL fluid Constituents

A total protein assay was modified for use on a Konelab 30 clinical chemistry analyzer (Thermo Clinical Labsystems Espoo, Finland). Total protein concentrations were determined with the Coomassie Plus Protein Reagent (Pierce Chemical, Rockford, IL) with a standard curve prepared with bovine serum albumin from Sigma-Aldrich (St. Louis, MO.). IL-4 protein was measured in BAL by ELISA using α-mouse IL-4 Biosource ELISA (Invitrogen, Carlsbad, California). Samples were run in duplicate and were read at 450 nm. IL-4 concentrations were determined by standard curve using the quadratic fit (R^2 ^= 0.998).

### Pulmonary Function Measurements

Pulmonary function changes to increasing concentrations of inhaled methacholine (Mch) were measured in mice using a 12-chamber whole-body plethysmograph system (Buxco Electronics, Troy, NY) on day 1, 4, 8, and 14 after influenza infection. Pressure signals were analyzed with BioSystem XA software (SFT3812, version 2.0.2.4, Buxco Electronics) to derive whole-body flow parameters that were used to calculate enhanced pause (Penh). Penh was used as an index of airflow obstruction, which has been correlated with changes in airway resistance [36]. After measuring baseline parameters for 7 min, an aerosol of saline or Mch in increasing concentrations (6.25, 12.5, and 25 mg/ml) was nebulized through an inlet of the chamber and mice were exposed for 10 minutes to each concentration. The recorded Penh values were averaged during the baseline periods and the 10 minute Mch challenges to obtain mean values for each event and were represented as change from the mean during the baseline period to the mean during each Mch challenge.

### Pulmonary Virus Quantification

Virus titers were expressed as the TCID_50 _per ml of lung homogenate. Briefly, the left lung was homogenized in 250 μl of DMEM containing 1 μg/ml of TPCK treated trypsin and 1% BSA. The homogenates were spun at 1000 × g to remove cellular debris and supernatants were used to determine tissue culture infectious dose that kills 50 percent of the cells (TCID_50_). Supernatants were plated on confluent MDCK cells in 96 well plates in log_10 _dilutions. After 3-5 days of incubation at 37°C, the cytopathic effect was observed and TCID_50 _was calculated using Reed-Muench method [37].

### Histopathology

Lung tissue samples were fixed in 4% paraformaldehyde and embedded in paraffin. Five μm thick sections were placed on Superfrost/plus slides (Fisher Scientific) and stained with H&E. The slides were evaluated by light microscopy at 10× and 40× objective by a veterinary pathologist (Dr. Mac Law) from NCSU College of Veterinary Medicine. Five to ten fields of at least 2 sections per animal and 2 animals per experimental group were evaluated. Tissue sections were photographed using an Olympus DP25 digital camera.

### Real Time PCR

Total RNA was extracted from lung tissue with TRIzol (Invitrogen, Grand Island, NY) as per the supplier's instructions. First strand cDNA synthesis and real-time RT-PCR were performed as previously described [38,39]. Genbank mRNA primers were IFN-γ NM_008337.1; IL-12p40 NM_008353.1; IL-4 NM_021283.1; IFN-β NM_008336.2; HO-1 NM_010442.1 purchased from Applied Biosystems (Foster City, CA). Expression changes were calculated using the relative quantification method. The housekeeping gene β actin was used as an endogenous reference to normalize target gene Ct values. Gene transcription was expressed as an n-fold difference relative to the control.

### Antioxidant Administration

A third experiment was designed to assess the effect of N-acetylcysteine (NAC) on the diesel enhanced influenza infection. Animals were treated with 320 mg/kg of NAC (Sigma-Aldrich, St. Louis, MO) in sterile saline intraperitoneally (i.p.) immediately before each inhalation exposure for the first four days as previously described [40].

### Glutathione levels

Perchloric acid (PCA, 60% solution) was added to lung homogenates to a final concentration of 3% and samples were stored at -80βC. Reduced glutathione (GSH) in the PCA supernatants were labeled with dansyl chloride by the method of [41] and analyzed by HPLC as described previously [42].

### Statistical Analysis

Data were pooled from the two initial exposure studies/experiments and were analyzed separately for the third anti-oxidant experiment, and were expressed as means ± SEM. Data generated from experiments were analyzed using nonparametric one-way ANOVA (Kruskal-Wallis test), followed by the Student Newman Kuehls comparison post hoc test. A value of P < 0.05 was considered to be significant.

## Results

### DE Chamber Concentrations

Table 1 shows a summary of the 13-day average exposure data for the control and (0.5 mg/m^3^) DE concentration. These target chamber concentrations, determined and adjusted based on continuous TEOM measurements, were achieved with relatively low variability either within a particular 4 hour exposure or between different days. Chamber particle concentrations determined gravimetrically from integrated filter samples (one 4 h sample per exposure day), agreed with the TEOM measurements within 15%. CO and NO_x _concentrations in the chambers averaged 12 and 17.6 ppm, respectively. SO_2 _concentrations were very low and below detection levels for the DE chamber. Particle number concentrations were relatively high and corresponded to particle size distributions (PSDs) with a well-established accumulation mode and little evidence of notable nuclei or coarse modes. Geometric median number and volume (assuming spherical particles) diameters were approximately 53 and 194 nm, respectively. It should be noted that the SMPS system (with long column) limited measurements to particles greater than approximately 15 nm, and a small increase in the number counts in channels less than 25 nm may indicate the presence of a small nuclei mode below the instrument's range. OC/EC wt ratios of 1.1 from both chambers indicated that approximately 52.4% of the DEP was comprised of organic carbon.

**Table 1:** Summary of concentrations and characteristics of the diesel exhaust particles and gases within the animal exposure chambers.^a^

*Constituent*	*Units*	*Exposure*
Particle mass concentration (TEOM)	μg/m^3^	500 ± 9
Particle mass concentration (filter)^b^	μg/m^3^	701 ± 16
Particle number concentration^c^	#/cm^3^	1.0 × 10^8^ ± 4.7 × 10^6^
Oxygen (O_2_)	%	19.7 ± 0.5
Carbon monoxide (CO)	ppm	12.0 ± 1.0
Nitrogen oxides (NO_x_)	ppm	17.6 ± 0.7
Sulfur dioxide (SO_2_)	ppm	>3.0
Number median D_p_^d^	nm	53 ± 2
Volume median D_p_^d^	nm	194 ± 2
OC/EC^e^	wt ratio	1.1 ± 0.1

^a^These data represent averaged results from the two repeat studies performed from 8/1-13/06 (13 days), 10/2-14/07 (13 days), and NAC study 2/11-19/08 (9 days). Tapered element oscillating microbalance (TEOM), O_2_, CO, and NO_x _data represent mean values from continuous measurements taken over all 35 exposure days ± SE.

^b^Filter data represent mean values from one measurement per day taken over 34 days of exposure ± SE.

^c^Particle number concentration data represent a mean value from 13 days of exposure ± SE.

^d^D_p _indicates particle geometric number and volume median diameters for 13 days of particle size distribution measurements ± geometric standard deviation. Note that volume information is calculated from number based mobility diameters and assume spherical particles.

^e^OC/EC (organic carbon to elemental carbon ratio) data represent mean values from one measurement per day taken over 13 days of exposure ± SE.

### Viral Quantification and Type I Interferon Production

**Figure 1:**
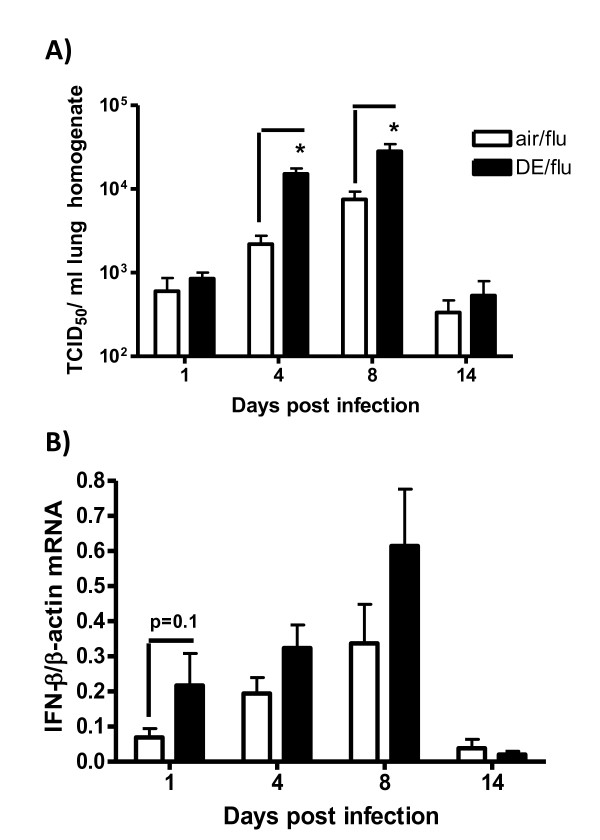
**Exposure to DE enhances influenza titers and IFN-β expression**. **A) **Viral titers were quantified in lung homogenates as TCID_50 _on day 1, 4, 8, and 14 p.i. **B) **IFN-β mRNA expression was quantified in lung RNA by RT-PCR. Values are normalized to β-actin and expressed as relative quantification. *significantly different from air exposed influenza infected mice (p < 0.05, n = 11).

The purpose of the study was to determine how DE affected the normal course of a sublethal viral infection, which is characterized by peak viral titers between day 4 and 8 p.i. accompanied by significant lung inflammation. Exposure to 0.5 mg/m^3 ^of DE during infection resulted in significantly greater levels of virus compared to air exposed mice at day 4 and 8 post infection while both the exposed and control animals essentially cleared the virus by 14 days (Figure 1A). Body weights were not significantly different between any of the treatment groups at any time point (data not shown).

Previous studies have demonstrated that chronic exposure of mice to DE resulted in increased viral titer in association with decreased lung IFN levels [10]. DE exposure alone did not affect IFN-β mRNA expression (data not shown), and although this cytokine was upregulated with virus infection with a peak at day 8 p.i, DE did not significantly affect the expression level (Figure 1B). Similarly, IFN-β mRNA was upregulated by the viral infection but the expression was not affected by exposure to DE either alone or in combination with virus (data not shown).

### Neutrophil Recruitment and Pulmonary Inflammation

**Figure 2:**
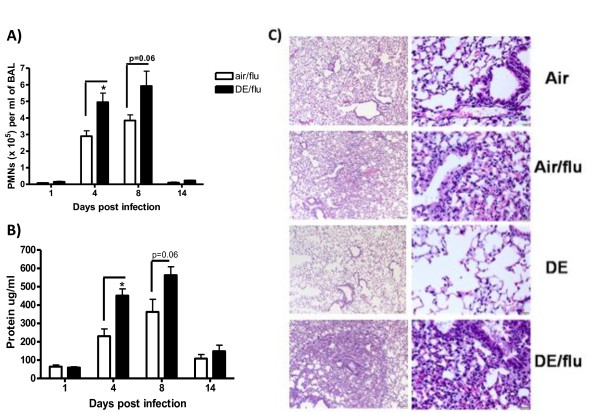
**Exposure to DE enhances influenza induced pulmonary inflammation**. BAL was obtained day 1, 4, 8, and 14 p.i. **A) **Neutrophil counts per ml of BAL. **B) **Protein concentration (μg/ml) in BAL. **C) **Pathology scores of mouse lung sections were stained with H & E and visualized and scored using light microscopy in tissues collected on day 4 p.i. Left column is representative of 100× magnification and right column is representative of 400× magnification. *significantly different from air exposed mice (p < 0.05, n = 8 for uninfected; n = 11 for influenza infected).

Differential cell counts from air or DE exposed mice with or without influenza infection were assessed in the BAL (Figure 2A). DE exposure alone caused a small but significant increase in PMNs at day 4, 8, and 14 (data not shown). The number of PMNs increased orders of magnitude more in the influenza infected animals, and at days 4 and day 8 p.i. these values were significantly higher in the DE exposed and infected animals at the day 4 timepoint and persisted at day 8 (p = 0.06) (Figure 2A). No residual PMNs were seen in the BAL at day 14 in any of the treatment groups.

The amount of protein in the BAL was also assessed as a marker of pulmonary edema. Influenza infection alone increased the amount of protein on day 4 and 8 p.i. with a return to baseline by day 14 p.i. (Figure 2B). Exposure to DE during infection caused an increase in the amount of protein in the BAL on day 4, which persisted to day 8, compared to air exposure (Figure 2B). DE alone did not significantly increase protein in the BAL at any time point (data not shown).

Histopathological examination showed patchy areas of mild interstitial inflammation in the lungs from influenza infected mice, and these lesions were judged to be more severe in the DE/flu animals as early as day 1, and even more prominently at day 4 p.i. (Figure 2C). Inflammation was characterized by presence of multifocal alveolar spaces that were thickened by edema and capillary congestion, and infiltrated with mild to moderate numbers of neutrophils, histiocytes, lymphocytes, and plasma cells. In the more severely affected areas of the influenza infected lungs, small amounts of lumenal exudate comprised of proteinaceous fluid with fibrin, cell debris, and often large (activated) foamy macrophages were present. In the diesel exposed groups (DE or DE/flu) the macrophages also contained multiple phagocytosed particles.

### Pulmonary Function

**Figure 3:**
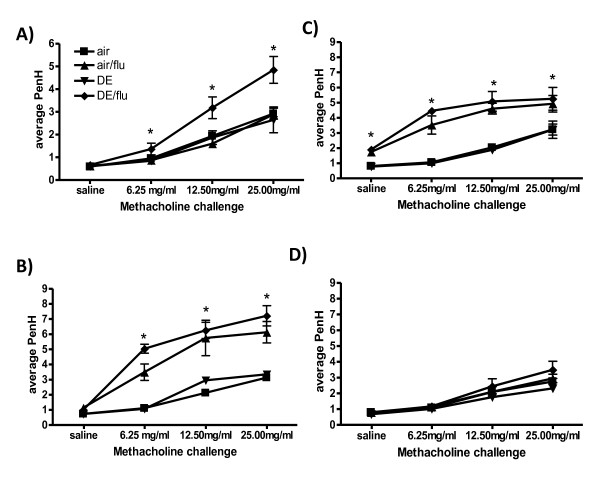
**Exposure to DE enhances influenza induced pulmonary responsiveness (PR)**. PR measured by Buxco systems at day 1, 4, 8, and 14 p.i., reported as PenH values with increasing doses of aerosolized methacholine. *significantly different from air and air/flu exposed mice (p < 0.05, n = 6).

Previous studies have shown that DE exposure [43] and influenza infection in mice [44] can independently increase pulmonary responsiveness (PR) to a methacholine challenge. Therefore it was of interest to examine the PR in the DE enhanced influenza infected mice as an indicator of altered pulmonary function and lung disease. DE exposure did not increase PR at any time point compared to air controls (Figure 3). Influenza infection alone significantly increased PR on day 4 and 8 p.i (Figure 3B and 3C). Mice exposed to DE during influenza infection had a significant increase in PR only at day 1 p.i. compared to influenza alone or DE exposure (Figure 3A). At day 4 and 8 p.i., the PR of DE/flu exposed mice was not significantly different from air/flu controls although both groups were significantly higher than responses in the air or DE alone exposed animals (Figure 3B and 3C). By 14 days all response curves returned to baseline levels with no differences between any of the groups (Figure 3D).

### Cytokine Expression in Lung

**Figure 4:**
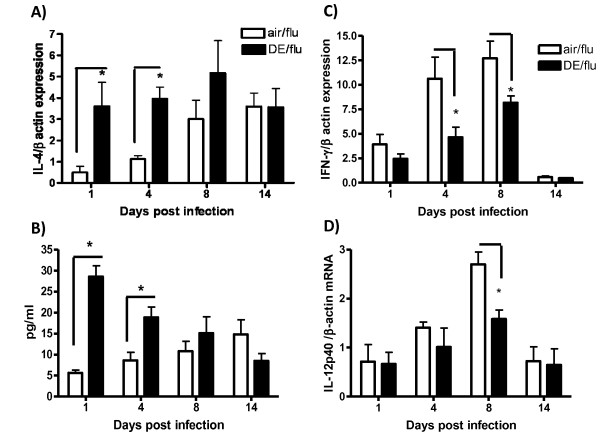
**Exposure to DE during an influenza infection increases the message of Interleukin 4 (IL-4) and decreases the expression of IFN-γ and IL-12p40 cytokines**. IL-4, IFN-γ and IL-12p40 gene expression was analyzed in lung RNA on day 1, 4, 8, and 14 p.i. IL-4 protein production was measured in BAL by ELISA at day 1, 4, 8, and 14 p.i. **A) **Levels of IL-4 mRNA were quantified in lung homogenates by real-time RT-PCR and normalized to levels of β-actin in mice exposed to air or DE during an influenza infection. **B) **Levels of IL-4 protein were measured in BAL. **C) **IFN-γ mRNA; **D) **IL-12p40 subunit mRNA. *significantly different from air or air/flu exposed mice (p < 0.05; n = 11 for influenza infected).

DE exposure has been reported to induce the production of Th2 cytokines [31] and since IL-4 decreases clearance of influenza [20], we hypothesized that the DE enhanced influenza infection was caused by an increase in IL-4. DE alone significantly increased IL-4 mRNA expression after 4, 8, and 14 days of exposure compared to the air controls (data not shown). Influenza infection alone also significantly increased IL-4 mRNA levels at day 8 and 14 p.i., while the DE exposure during influenza infection resulted in significantly higher IL-4 mRNA expression at days 1 and 4 p.i. compared to air exposed infected mice (Figure 4A). This was also reflected in protein expression of IL-4 in BAL (Figure 4B). IL-13 message was also measured in the lung tissue of all treatment groups and no differences were observed indicating that this response maybe specific to IL-4 (data not shown).

Th1 cytokines including IL-12 and IFN-γ are required to clear influenza infection in mice [45]. DE exposure alone did not alter expression of either cytokine at any necropsy time point compared to air control (data not shown). Influenza infection significantly increased IFN-γ and IL-12p40 at day 1, 4, and 8 p.i before returning to baseline at day 14 (Figure 4C and 3D) and these increases were significantly reduced with concomitant exposure to DE at the day 4 and 8 timepoints

### Effects of NAC on Glutathione and Hemeoxygenase-1(HO-1)

**Figure 5:**
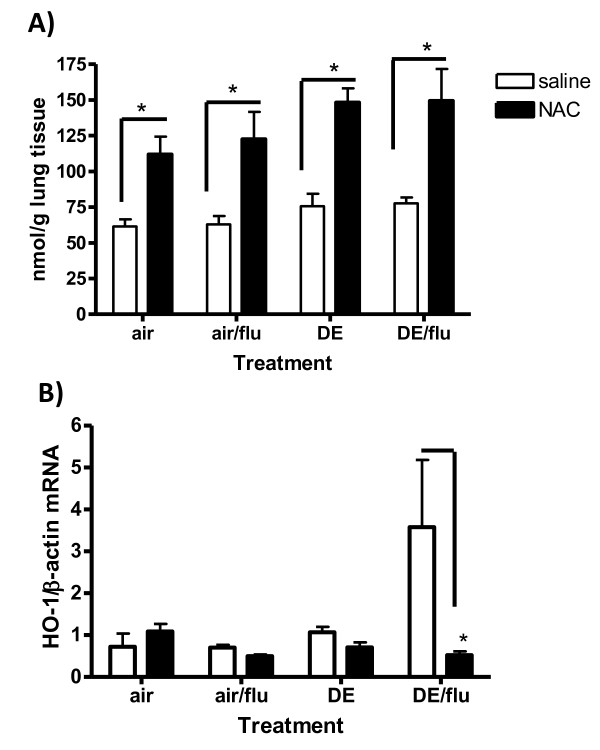
**Antioxidants can increase glutathione (GSH) and decrease hemeoxygenase 1 (HO-1) expression during DE enhanced influenza infection**. **A) **Levels of GSH were quantified in PCA lung homogenates by HPLC and expressed as nmol of GSH per gram of lung tissue. Graph is representative of day 4 p.i. **B) **Levels of HO-1 mRNA were quantified in lung homogenates by real-time RT-PCR and normalized to levels of β-actin in mice exposed to air on day 4 p.i. (p < 0.05, n = 6).

To determine if the increase in viral disease parameters associated with DE exposure was a result of oxidative stress, mice were injected i.p. with 320 mg/kg NAC or vehicle 2 hours before each exposure as previously described [40]. One of the mechanisms for NAC-mediated decrease in oxidative stress is to upregulate the amount of reduced glutathione (GSH) available for detoxifying reactive species [46]. Mice given NAC before all treatments had an increase in lung GSH levels and this was significant in mice exposed to DE or DE/flu at day 1 and 4 p.i. (Day 4 represented in Figure 5A). HO-1 mRNA expression after 1 or 4 days of DE exposure was also elevated in the mice exposed to DE during influenza infection and this was ameliorated with NAC treatment. (Day 4 represented in Figure 5B).

### Effect of NAC on Viral Titers

**Figure 6:**
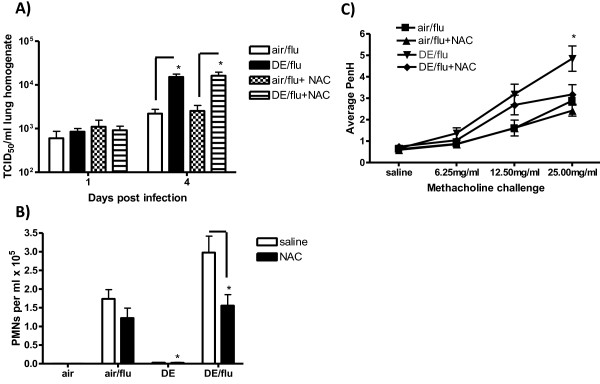
**Antioxidants have no effect on DE enhanced influenza titers but decrease DE enhanced pulmonary inflammation**. **A) **Viral titers were quantified in lung homogenates as TCID_50 _on day 1 and 4 p.i. *significantly different from air/flu (p < 0.05, n = 6). **B) **Neutrophil counts per ml of BAL on day 4 p.i. **C) **PR measured by Buxco systems at day 1 p.i. reported as PenH values with increasing doses of aerosolized methacholine. *significantly different from saline controls (p < 0.05, n = 6).

Our previous studies have shown that an increase in GSH in an *in vitro *DE/flu model decreased the amount of influenza virus attaching to the epithelium [12]. Whole lung homogenates were evaluated for TCID_50 _titers to determine if NAC decreased the DE enhanced viral titers at day 4 p.i. As before, DE exposure increased viral titers on day 4 p.i. compared to air/flu mice (Figure 6A), however unlike the GSH and HO-1 results, this effect was not abrogated by NAC treatment (Figure 6A).

### Effect of NAC on Inflammation and Pulmonary Function

To determine if NAC treatment decreased pulmonary inflammation, the biomarkers that were previously measured were assessed. Neutrophil counts that were significantly increased with influenza infection and DE exposure were decreased at day 4 p.i. in mice treated with NAC (Figure 6B) and a similar pattern was seen for BAL protein (data not shown). As before, DE exposure increased PR in mice infected with influenza on day 1 p.i. (Figure 6) and this was decreased PR to baseline levels in the NAC treated animals (Figure 6C).

### Effect of NAC on Cytokine Expression

**Figure 7:**
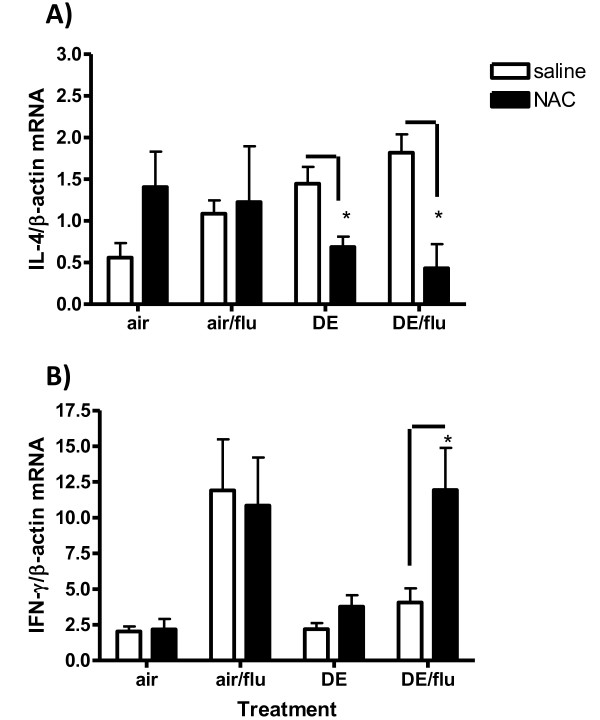
**Antioxidants decrease Interleukin 4 (IL-4) and increase the expression of IFN-γ during a DE-enhanced influenza infection**. IL-4 and IFN-γ gene expression was analyzed in lung RNA on day 4 p.i. **A) **Levels of IL-4 mRNA were quantified in lung homogenates by real-time RT-PCR and normalized to levels of β-actin in mice exposed to air on day 4 p.i. **B) **IFN-γ on day 4 p.i. *significantly different from saline control (p < 0.05, n = 6).

As noted in figure 4A, the DE exposure augmented expression of IL-4, at day 4 p.i., and this effect was abrogated by pre-treatment with NAC (Figure 7A). A NAC-related reduction in IL-4 was also seen with mice exposed to DE alone. No differences in IL-4 expression were seen between NAC treated and untreated mice infected with influenza but not exposed to DE, while NAC alone increased IL-4 expression in untreated mice (air alone) as has been previously reported [47]. NAC did not alter IL-13 expression in lung tissue of mice at day 1 or 4 p.i. in any of the treatment groups (data not shown).

No difference was seen in IFN-γ mRNA with NAC in mice exposed to air or influenza alone (Figure 7B). However, on day 1 p.i., (and 4 p.i., data not shown) the NAC-treated mice exposed to DE or DE/flu had increased expression of IFN-γ compared to saline controls (Figure 7B). No differences were seen with IL-12p40 expression at these early time points although no effect would have been expected until day 8 when a late DE- related reduction occurred (Figure 4C).

## Discussion

Air pollution exposure has been linked to exacerbation of multiple pulmonary diseases as well as increased severity of viral infections. In many cases, these effects have been associated with concomitant increases in susceptibility to respiratory infection (reviewed in [6]). Numerous experimental studies have demonstrated a decrease in a broad range of innate and adaptive host defenses following inhalation exposure to both single agents or complex mixtures such as cigarette smoke and car exhaust [48]. While most reports were designed to assess how pollutant exposure affected a subsequent pathogenic challenge, the present study followed the course of viral proliferation during an ongoing exposure to diluted diesel exhaust. The results show that inhalation exposure to DE promoted viral proliferation in the lung in association with altered immune signaling and increased markers of lung disease. Administration of the antioxidant NAC reduced many of the DE-enhanced signaling and pathological changes but did not affect the course of virus infection.

There are myriad mechanisms by which DE can potentiate a respiratory infection. The toxic particles and gases may singly or in combination have a multitude of effects including reducing mucociliary clearance [49], decreasing alveolar macrophage function [50], diminishing production of antiviral defenses [13] as well as having more broad systemic immuntoxicity affecting both the myeloid and lymphatic systems [10]. Recently we demonstrated that exposure to DE decreased expression and production of host defense molecules including surfactant protein A (SP-A), SP-D, and clara cell secretory protein (CCSP) [51], and this was associated with increased proliferation of influenza virus in both in vitro [52] and vivo systems [13,53-55]. In addition to these first line defenses, a delicate balance between Th1 and Th2 immunity is required for optimum recovery from influenza virus infection. The data presented here clearly demonstrate that the DE caused significant increases in IL-4 and a concomitant decrease in IFN-γ and IL-12 in association with increased viral-induced disease. However, it is unclear whether increased viral-induced disease (PMNs, inflammation) in DE enhanced influenza infection is responsible for adequate clearance, since viral persistence was not seen. Previous studies have noted that the presence of pulmonary infiltrates such as PMNs are essential for viral clearance [56,57], however excessive neutrophilia and inflammation has been cited to be detrimental to the host during an influenza infection [58].

It is generally understood that both Th1 and Th2 cytokines are needed at specific times during viral infection to stimulate both cell-mediated and antibody mediated immune responses, as well as immunological memory [59,60]. Despite this dual requirement, inappropriate production of Th2 cytokine IL-4 has been reported to increase severity of lung disease during influenza infection [20,61], and IL-4 production during viral infections diminishes the number of MHC class I-restricted T cells and polarizes CD4+ T cells away from an IFN-γ dominated response [21]. Moreover, mice that lack functional IL-4 genes clear sub-lethal doses of influenza more efficiently [62] while IL-12 or IFN-γ deficient mice do not [19,63]. IFN-γ not only stimulates antiviral mediator expression and increases antigen presentation to T lymphocytes, but also activates NK cells, which facilitate viral clearance particularly during the early stages of an influenza infection [64]. In the present study, DE exposure either alone, or during influenza infection significantly increased the expression of IL-4 in association with a later decrease in IFN-γ and IL-12p40 expression. However, despite the polarization towards a Th2 phenotype, IL-13 levels were not different in this model (data not shown) suggesting that DE enhanced influenza infection may specifically increase Th2 cytokines induced in influenza such as IL-4. Thus, early polarization towards an increased IL-4/IFN-γ ratio could be a mechanism behind air pollution-enhanced influenza virus levels and the observed increase in lung disease. Further studies are needed to characterize how air pollutants can induce IL-4 during respiratory infections.

Viral infections in the lung are normally associated with epithelial injury, inflammation, increased mucus production and sometimes airway hyperreactivity in response to inhaled agonists [44,65,66]. In order to measure these pathophysiological processes we first tested the animals' responsiveness to methacholine aerosol in a whole body plethysmograph prior to assessing pulmonary inflammation in the lung fluid, as well as by standard histopathology. The results showed that the pulmonary responsiveness mirrored the inflammatory wave to the virus as expected. Interestingly however, higher PR was only observed at 1 day in the DE/flu animals in the absence of significant inflammation and increased PR was not observed at later timepoints. Since whole body plethysmography is a rather crude indicator of pulmonary function with the unitless enhanced pause (PenH) value being derived from thoracic expansion and air flow through the nose [67], other more traditional measures of airway resistance are needed to confirm this results. It is known that influenza infection causes airway hyperreactivity [44,66] and also, that this may also be found in the absence of inflammation through for example neurogenic pathways [68]. Furthermore, the infection was performed by oropharyngeal aspiration and likely did not cause significant alterations in nasal architecture and the DE exposure alone did not result in significant PR changes. Taken together the results suggest that altered pulmonary function occurred early on as a result of synergism between virus and DE, although as the effect progressed through day 4 and day 8 the enhanced responsiveness was just as strong with virus alone.

It has been hypothesized that the increase in Th2 phenotype by DE is a result of ROS and oxidative stress releasing nuclear factor-erythoid 2 (NF-E2) - related factor 2 (Nrf2) which initiates expression of phase II enzymes such as HO-1 [69]. The expression of these enzymes drives the immune system to produce more of a Th2 cytokine profile [70] that can be blocked by the administration of the thiol specific antioxidant NAC [40,43] that is converted into GSH [33,41]. This was reflected in the present study by significantly elevated concentrations of GSH in mice treated with NAC and exposed to either DE or DE/flu along with a corresponding decrease in HO-1 expression. Although, administration of NAC blocked the DE-enhanced pulmonary inflammation and PR to levels comparable to the air/flu control, the treatment did not affect viral titers or associated pathology. This would indicate that oxidative stress is not the only mechanism by which DE exposure enhances the influenza virus replication although it clearly contributed to the resulting pathology. Administration of NAC also reversed the polarization from an IL-4 dominated response at day 1 and 4 p.i. in DE/flu mice to a more IFN-γ dominated profile on day 4 p.i. This re-polarization could be explained by increasing GSH concentrations, which have previously been shown to interfere with IL-4 production and favor a Th1 phenotype [71]. Likewise, GSH depletion has been reported to shift the immune system towards an IL-4 dominated response [70] by changing dendritic cell (DC) characteristics through pulmonary inflammation and oxidative stress [72]. Previous studies have shown that DEP co-incubated with DCs induce pro-oxidative responses that inhibit stimulation of Th1 CD4^+ ^T cells [69]. All of this taken together indicates that DE-induced oxidative stress during an influenza infection polarizes the immune system, possibly by altering the phenotype and maturation of DCs.

In summary we have shown that oxidative stress induced by DE exposure during an influenza infection resulted in increased viral titers and markers of lung disease. The DE exposure promoted an IL-4 dominated response that could be reversed by thiol antioxidants, which may provide therapeutic strategies for at risk populations. While the temporary reduction in protective immunity did not affect eventual clearance of the virus, the increased severity of infection and duration of symptoms would be associated with greater morbidity as well as potentially increased susceptibility to secondary bacterial infections. In addition, viral infections are the major cause for exacerbation of allergic airway disease. We have previously reported that exposure to DEP increases virus-induced allergic inflammation in mice [73]. Based on the data shown here, it is plausible that the increased production of IL-4 served to both reduce anti-viral immunity while promoting the development of allergic airway disease. Given the significant influenza-related morbidity and mortality worldwide and the growing incidence of allergic asthma, it is important to further understand the interactions between pollution exposure and pulmonary immunity associated with respiratory virus infections, and the interplay with other forms of lung disease.

## Abbreviations

**APC: **antigen presenting cells; **BAL: **bronchoalveolar lavage; **DE: **diesel exhaust; **DEP: **diesel exhaust particles; **GSH: **reduced glutathione; **HO-1: **heme-oxygenase 1; **IL: **interleukin; **IFN: **interferon; **NAC: **n-acetylcysteine; **PFU: **plaque forming units; **PM: **particulate matter; **PMN: **polymorphonuclear leukocyte; **PR: **pulmonary responsiveness; **ROS: **reactive oxygen species; **RSV: **respiratory syncytial virus **TCID_50_: **tissue culture infectious dose; **TPCK: **L-(tosylamido-2-phenyl) ethyl chloromethyl ketone.

## Competing interests

The authors declare that they have no competing interests.

## Authors' contributions

KG carried out animal studies as well as immuno and molecular assays, study design, statistical analysis and drafted manuscript. EB helped with performing animal studies and assays on tissue endpoints. CK and QK performed analysis of diesel exhaust and helped with study design. IJ helped with study design and data analysis as well as an intellectual contributor. WL is responsible for diesel inhalation facility and intellectual contributions. MIG is the PI on the study and is also responsible for study design, manuscript preparation and data analysis. All authors have read and approved the final manuscript.
